# Checklist of coastal fishes from Cabo Verde Archipelago

**DOI:** 10.3897/BDJ.13.e148234

**Published:** 2025-06-16

**Authors:** Luís M D Barcelos, Rui Freitas, João Pedro Barreiros

**Affiliations:** 1 ce3c - Centre for Ecology, Evolution and Environmental Changes & CHANGE - Global Change and Sustainability Institute, Angra do Heroísmo, Portugal ce3c - Centre for Ecology, Evolution and Environmental Changes & CHANGE - Global Change and Sustainability Institute Angra do Heroísmo Portugal; 2 University of the Azores, Faculty of Agricultural Sciences and Environment, Angra do Heroísmo, Portugal University of the Azores, Faculty of Agricultural Sciences and Environment Angra do Heroísmo Portugal; 3 ISECMAR - Universidade Técnica do Atlântico, Mindelo, Cabo Verde ISECMAR - Universidade Técnica do Atlântico Mindelo Cabo Verde; 4 IUCN - International Union for the Conservation of Nature, Hong Kong, Hong Kong IUCN - International Union for the Conservation of Nature Hong Kong Hong Kong

**Keywords:** ichthyo-diversity, ichthyofauna distribution, new records, regional inventory, species inventory verification, tropical east Atlantic

## Abstract

**Background:**

Taxonomic and geographic misattributions in biodiversity inventories remain a pressing issue in biogeographical research, particularly in regions with overlapping or similar place names. The Republic of Cabo Verde (also known as Cabo Verde Islands) and the Cape Verde Peninsula (Senegal) exemplify this challenge, where historical and recent studies have struggled to provide accurate species distributions due to unverified, erroneous and ambiguous records. This underscores the necessity of comprehensive, reliable datasets to delineate species occurrences across these distinct geographic areas.

**New information:**

This study provides a rigorously verified inventory of coastal fish species occurring within the Exclusive Economic Zone (EEZ) of the Republic of Cabo Verde, focusing on depths between 0 and 200 metres. By delineating the faunal composition specific to Cabo Verde, this work addresses the recurrent confusion with species lists referencing the Cape Verde Peninsula (Senegal).

A total of 393 species, distributed among 125 families and 40 orders, is documented, offering an invaluable resource for refining biodiversity assessments and providing information forconservation strategies in this biogeographically unique region. Two species, *Thalassomanewtoni* (Osório, 1891) and *Diodoneydouxii* Brisout de Barneville, 1846, are reported for the first time from Cabo Verde in this paper.

## Introduction

Checklists are important records of local biodiversity, furnishing baseline data to support conservation initiatives and ecological investigations, as they provide a structured approach to cataloguing and tracking biodiversity (Lim et al. 2024). Wirtz et al. (2013) produced the first comprehensive, annotated and illustrated checklist of the coastal fishes of the Cabo Verde Islands, which includes previously recorded species, new non-records, doubtful species, invalid records and species for which additional data are required.

These tools enhance our understanding of species’ distribution and abundance patterns, which is pivotal for implementing effective management and conservation strategies (Droege et al. 2008, Reyserhove et al. 2020, Lo Brutto 2023). Furthermore, they serve as foundational resources for improving local Red Lists and contribute to broader conservation biology efforts and also providing a standardised format for sharing taxonomic information, which is essential for collaborative conservation (Droege et al. 2008, GBIF 2017).

**Figure 1.**
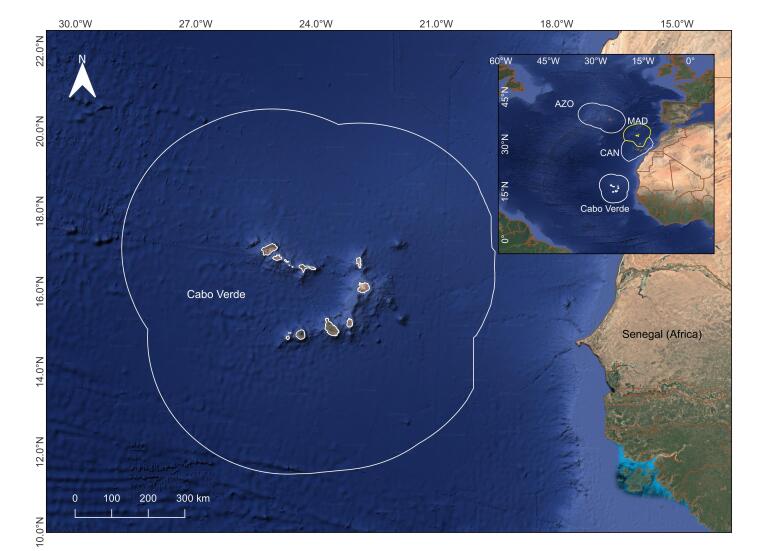
Cabo Verde with EEZ (white line). Inserted - Cabo Verde locations in eastern tropical Atlantic in relation to the other Macaronesian archipelagos (AZO: Azores, MAD: Madeira, CAN: Canary Islands).

Cabo Verde is an archipelagic country located in the eastern central Atlantic (Hoving et al. 2020), west of Senegal (Fig. 1). This volcanic archipelago comprises ten main islands and several smaller islets, characterised by steep shorelines that rise from ocean depths exceeding 3,000 metres (Wenzel et al. 2020).

The archipelago lies at the eastern boundary of the North Atlantic subtropical gyre and at the southern limit of the Canary Current, while also being influenced by the North Equatorial Counter-Current. The climate is tropical, with two alternating seasons: a mild period (December to June) and a warm season (July to November) (Peña-Izquierdo et al. 2012). As in other islands within this ecoregion, Cabo Verde’s weather patterns are influenced by the Atlantic Ocean and the subtropical high-pressure system. The distances, habitat heterogeneity and bathymetric features amongst island groups result in the physical isolation of a naturally fragmented landscape, shaping the distribution patterns of the archipelago’s marine fauna (Medina et al. 2007).

The Sahelian Upwelling acts as a cryptic barrier to the dispersal of nektonic marine species between Cabo Verde and the African mainland, as indicated by the differences in ichthyofauna observed by Wirtz (2009) and Wirtz (2012) between Ngor Island (Senegal) and the Cabo Verde Islands. In fact, Cabo Verde shows stronger marine faunal affinities with the Guinean Region and the Canary Islands than with the north-west African coast (Morri et al. 2000, Wirtz et al. 2013).

The Cabo Verde Islands exhibit a much higher degree of coastal fish endemism than the other Macaronesian archipelagos (i.e. the Azores, Madeira, Selvagens and Canary Islands) and, in terms of the number of endemic taxa, Cabo Verde more closely resembles the islands in the Gulf of Guinea than the north-west African coast (Wirtz et al. 2013). From the perspective of marine community structure and ichthyogeography, Cabo Verde diverges substantially from the other Macaronesian islands (Brito et al. 1999, Morri et al. 2000, Brito et al. 2006).

Historically, fish lists for Cabo Verde have been compiled from multiple sources, many of which inadvertently included data pertaining to the Cape Verde Peninsula (Senegal) rather than the archipelago. An updated, comprehensive list was, therefore, needed for the coastal species inhabiting Cabo Verde’s waters. This paper compiles the most recent data available on the archipelago’s fish diversity. The ichthyological diversity of the islands is recognised as unique, with several endemic species (Table 1) (Reiner 2005, Wirtz et al. 2013, Freitas 2014).

## General description

### Purpose

Using published works - Brito et al. (1999), Fricke et al. (2010), Hanel et al. (2010), Wirtz et al. (2013), Freitas (2014), Wirtz (2014), González et al. (2016), Freitas et al. (2018), González et al. (2018), Schliewen et al. (2018), Freitas et al. (2019a), Freitas et al. (2019b) amongst others, we have produced this list of species.

### Additional information

We define coastal species as those occurring at depths of up to 200 metres. This definition includes species that may be more commonly found below this depth, but which are known to occur, at least occasionally, in shallower waters.

Other species are possibly present, but have not yet been detected, such as *Isistius* spp. (see Barcelos et al. 2024). The Cabo Verde Islands lie within the distribution range of both species of this genus and interactions between these species and cetaceans in Cabo Verdean waters have already been documented (Wenzel and Suárez 2021).

## Project description

### Title

Checklist list of Coastal fishes of Cabo Verde.

### Personnel

Evandro P Lopes acted as collection curator and database management.

### Study area description

Located in the eastern central Atlantic, 570 km west of Ngor Island (Senegal, West African coast) the Cabo Verde archipelago is composed of ten islands and eight islets formed by rock accumulation arising from volcanic eruptions from a hotspot under submarine platforms. The total land area of 4,033 km^2^ with ~ 965 km of coastline which is believed to have formed between ~ 3 to 15.8 Ma. The insular marine platform (< 200 m) surrounding the islands covers approximately 1,900 km^2^, representing only 0.2% of the total Cabo Verde Exclusive Economic Zone (EEZ). The total EEZ area of Cabo Verde accounts for 796,000 km^2^ excluding the insular shelf area (isobaths depth < 200 m).

### Design description

State-of-the-art literature review.

## Sampling methods

### Study extent

Literature review including all published and confirmed occurrences focusing on updated and recent publications.

### Sampling description

State-of-the-art literature review.

### Step description

Literature review, fieldwork (sampling), occurrence confirmation.

## Geographic coverage

### Description

Surrounding area of the Cabo Verde archipelago, spreading from the country's Exclusive Economic Zone (ZEE).

### Coordinates

14 and 18 Latitude; -26 and -22 Longitude.

## Taxonomic coverage

### Description

This list comprises all shallow water (down to 200 m) records of both elasmobranch and actinopterigyans so far confirmed for the geographical area of Cabo Verde. The results presented are updated and subject to changes as all checklists are dynamic.

This checklist (Table 2) comprises 393 species across 38 orders and 125 families (Table 3). Species are listed alphabetically within families, following the taxonomy proposed by Fricke et al. (2025) and Van der Laan et al. (2025).

### Taxa included

**Table:** 

Rank	Scientific Name	Common Name
class	Elasmobranchii	Elasmobranchs (Sharks and Rays)
class	Actinopteri	Ray finned fish

## Temporal coverage

### Notes

This work does not define a specific temporal scope, as it compiles data from historical inventories and faunistic records. By synthesising information across a broad temporal range and integrating it with current knowledge of Cabo Verde’s coastal ichthyofauna, the study presents a comprehensive overview of present understanding of coastal fish assemblages.

## Usage licence

### Usage licence

Creative Commons Public Domain Waiver (CC-Zero)

### IP rights notes

This work is licensed under a Creative Commons Attribution (CC-BY 4.0) License.

## Data resources

### Data package title

Checklist list of Coastal fishes of Cabo Verde

### Resource link

https://www.gbif.org/dataset/ecf78ae1-c4dc-48cc-9ff3-bb77d46a8e76

### Alternative identifiers

http://ipt.gbif.pt/ipt/resource?r=cabo_verde_coastal_fishes

### Number of data sets

1

### Data set 1.

#### Data set name

Checklist list of Coastal fishes of Cabo Verde

#### Data format

Darwin Core

#### Character set

UTF-8

#### Download URL

http://ipt.gbif.pt/ipt/archive.do?r=cabo_verde_coastal_fishes&v=1.1

#### Description

List of coastal fish species (0-200 metres deep) with confirmed updated occurrences in the Cabo Verde archipelago.*^1^

This list includes 393pecies belonging to the classes **Elasmobranchii** and **Actinopteri**. Taxonomic and phylogenetic ordering follows Fricke et al. (2025) and Van der Laan et al. (2025) down to the family level, with genera and species listed alphabetically.

For certain species, we were unable to confirm their occurrence in the archipelago or existing records were deemed dubious or erroneous. These taxa are listed in Table 5, along with the sources that previously reported them for Cabo Verde.

**Data set 1.** 

Column label	Column description
id	Life Sciences Identifier: World Register of Marine Species: Taxon ID.
taxonID	Life Sciences Identifier: World Register of Marine Species: Taxon ID.
acceptedNameUsageID	Life Sciences Identifier: World Register of Marine Species: Taxon ID.
parentNameUsageID	Parent Name: Life Sciences Identifier: World Register of Marine Species: Taxon ID.
parentNameUsage	parentNameUsage.
scientificName	scientificName.
Kingdom	Kingdom.
phylum	phylum.
class	class.
order	order.
family	family.
genus	genus.
specificEpithet	specificEpithet.
taxonRank	Rank of the taxon.
scientificNameAuthorship	Authorship of the scientific name.

## Additional information

Coastal fishes from Cabo Verde (Eastern Central Atlantic).

### Analysis and discussion

**Figure 2.**
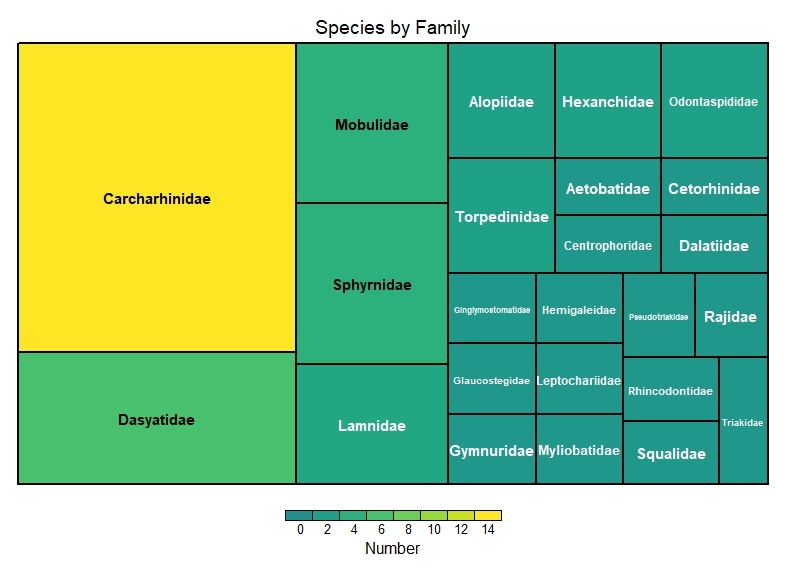
Elasmobranchii number of species by family.

**Figure 3.**
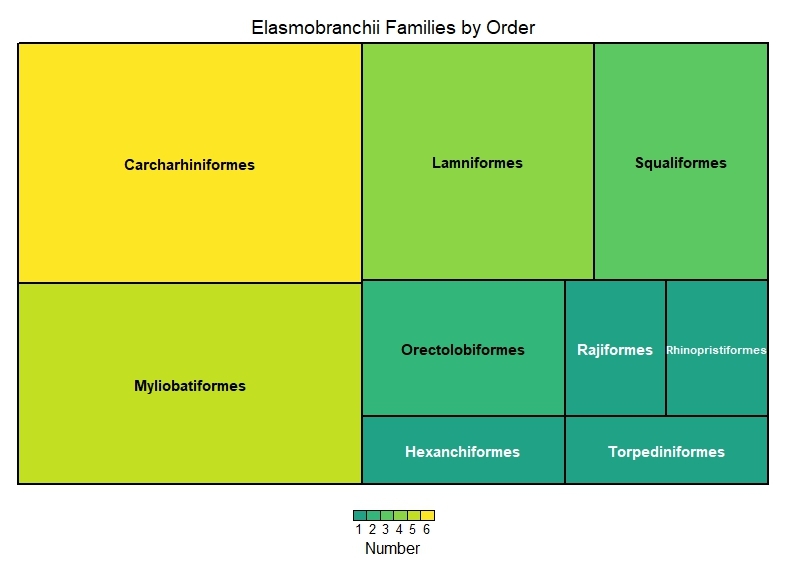
Elasmobranchii number of families by order.

**Figure 4.**
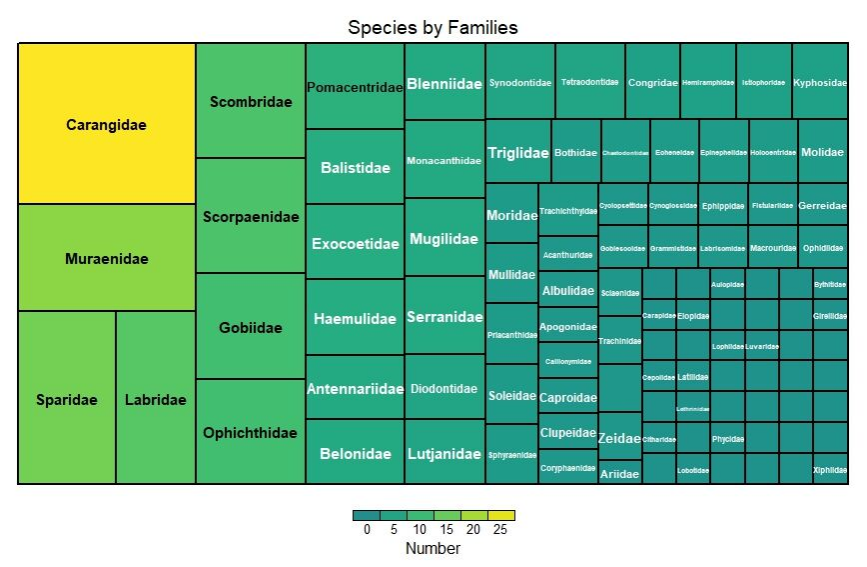
Actinopteri number of species by family.

**Figure 5.**
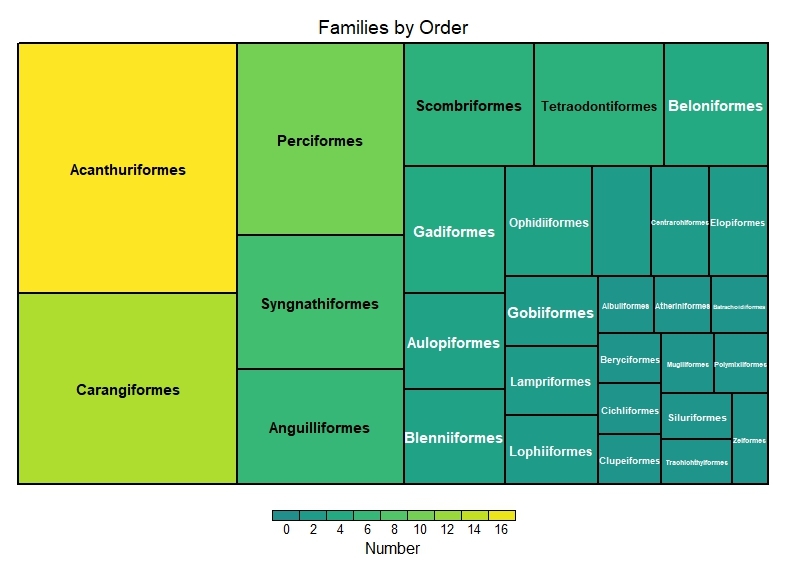
Actinopteri family numbers by orders.

This dataset encompasses the classes Elasmobranchii and Actinopteri (Table 4). Elasmobranchii is represented by 10 orders, 22 families and 50 species (Figs 2, 3), while Actinopteri comprises 29 orders, 103 families and 343 species (Figs 4, 5).

**Figure 6.**
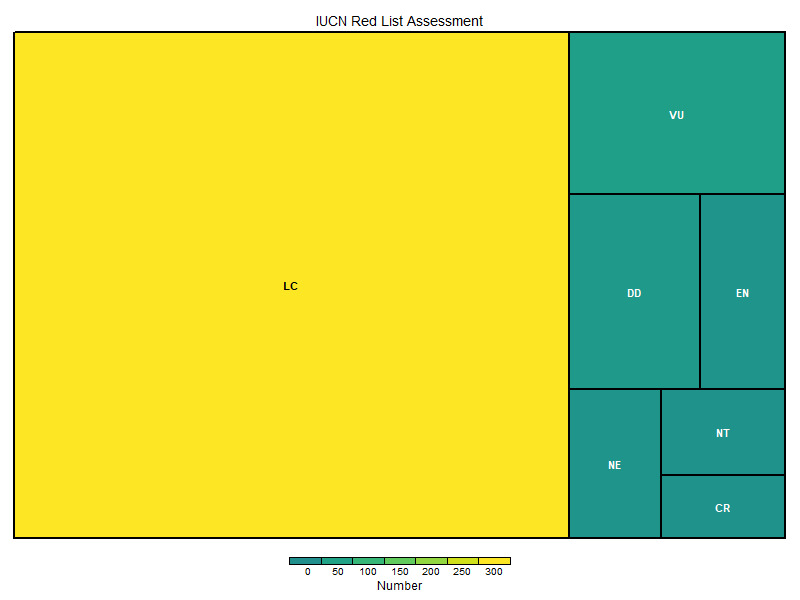
IUCN Accessemnts by numbers. NE - Not Evaluated, DD - Data Deficient, LC - Least Concern, NT - Near Threatned, VU - Vulnerable, EN - Endangered, CR - Critically Endangered.

Regarding conservation status (Fig. 6) and according to the IUCN Red List (IUCN 2024), the majority of species were assessed as of Least Concern. However, approximately 15% fall into threatened categories, including Vulnerable, Endangered or Critically Endangered.

Our species list is based primarily on the compilation by Wirtz et al. (2013), as it includes critical commentary on species occurrences. In some cases, we also incorporated earlier records not mentioned in Wirtz et al. (2013), as well as more recent publications that confirmed or expanded upon previous observations. Additionally, we considered selected ‘grey literature’ sources, which provide relevant information on species presence and photographic documentation, such as Mascarenhas (2022).

Wirtz et al. (2013) Wirtz et al. (2013) Mascarenhas (2022)

Since we followed the phylogenetic and taxonomic arrangement proposed by Fricke et al. (2025) and Van der Laan et al. (2025) Eschmeyer's Catalogue of Fishes, with orders and families sorted accordingly and species listed alphabetically. Scientific names and taxonomic validity were also based on Eschmeyer’s taxonomy, which may differ from the nomenclature used in some of the referenced literature. This is exemplified by *Kyphosus* spp. and *Mullusafricanus*, both considered valid species in Eschmeyer, whereas *Mullusargentinaeafricanus* is retained as a subspecies by Uiblein et al. (2024), who argues that the available evidence is insufficient to justify its elevation to species level. Another case is *Uraspissecunda*, recorded for Cabo Verde by Wirtz et al. (2013), yet considered a synonym of *Uraspishelvola* (Forster, 1801) in Eschmeyer. Further discussion on this taxonomic decision is provided by Carpenter and De Angelis (2016b).

In exceptional cases where new species records have not yet been formally published, we relied on first-hand observations reported to one of the authors (Rui Freitas - RF). These records were accepted when the observer’s expertise was deemed sufficiently reliable to validate the occurrence. On two occasions, we confirmed species presence through museum specimens, namely *Sauridabrasiliensis* Norman, 1935 and *Coelorinchuscaelorhincus* (Risso, 1810); however, we were unable to locate the original publications corresponding to these records.

We here validate the presence of *Thalassomanewtoni* (Osório, 1891), which was initially mentioned by Reiner (2005), but listed as a “mistaken record” in Wirtz et al. (2013). Our confirmation is based on a video recording (Fig. 9), published on YouTube by @snorkeeb.716 in June 2018 on Tarrafal (Santiago Island) and sent to RF by Juan Torres.

**Figure 8.**
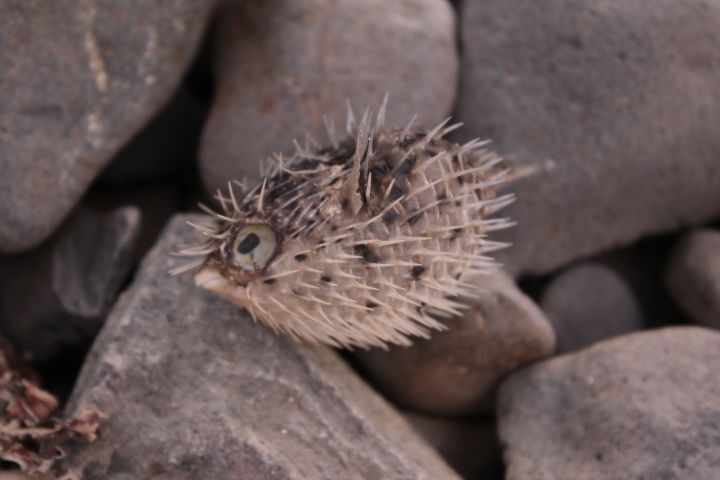
A photograph taken by Samir Martins in 2016 documents the presence of *Diodoneydouxii* Brisout de Barneville, 1846, at Ponta Antónia, Boa Vista Island (Cabo Verde).

Another noteworthy case is that of *Diodoneydouxii* Brisout de Barneville, 1846 (Fig. 8), which was found washed ashore at Ponta Antónia (Boa Vista Island). This specimen was one of 25 species stranded during the same event. To our knowledge, the only record of this occurrence was published in a local newspaper by RF. As far as we are aware, this constitutes the first confirmed and documented occurrence of the species within the Cabo Verde archipelago.

## Figures and Tables

**Figure 7.**
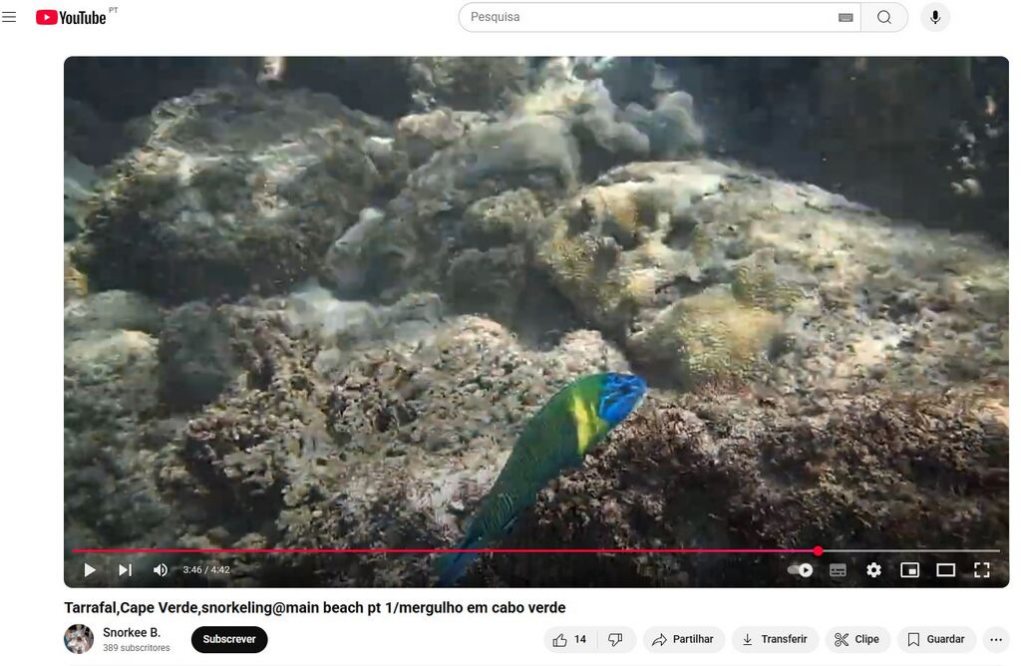
A frame captured from a video recorded by @snorkeeb.716 in June 2018, at Tarrafal (Santiago Island, Cabo Verde), at timestamp 3 minutes and 45 seconds, clearly shows a specimen of *Thalassomanewtoni* (Osório, 1891).

**Figure 9.** The video, recorded by @snorkeeb.716 in June 2018 at Tarrafal (Santiago Island, Cabo Verde), shows a specimen of *Thalassomanewtoni* (Osório, 1891) at the 3 minutes and 45 seconds mark.

**Table 1.** Endemic coastal species (n = 23) of Cabo Verde, belonging to 13 families and seven orders.

**Order**	**Family**	**Scientific name**
Rajiformes	Rajidae	*Rajaherwigi* Krefft, 1965
Gobiiformes	Gobiidae	*Gobiusateriformis* Brito & Miller, 2001
		*Gobiussalamansa* Iglésias, Frotté & Sellos, 2015
		*Gobiustetrophthalmus* Brito & Miller, 2001
		*Marcelogobiusjanetarum* (Schliewen, Wirtz & Kovačić 2018)
		*Mauligobiusnigri* (Günther, 1861)
Carangiformes	Soleidae	*Pegusacadenati* Chabanaud, 1954
Beloniformes	Belonidae	*Platybelonelovii* (Günther, 1866)
Mugiliformes	Mugilidae	*Chelonbispinosus* (Bowdich, 1825)
Blenniiformes	Pomacentridae	*Chromislubbocki* Edwards, 1986
		*Similiparmahermani* (Steindachner, 1887)
	Gobiesocidae	*Apletodonbarbatus* Fricke, Wirtz & Brito, 2010
	Blenniidae	*Microlipophryscaboverdensis* (Wirtz & Bath, 1989)
		*Parablenniussalensis* Bath, 1990
		*Scartellacaboverdiana* Bath, 1990
	Labrisomidae	*Malacoctenuscarrowi* Wirtz, 2014
Perciformes	Pinguipedidae	*Parapercisatlantica* (Vaillant, 1887)
Centrarchiformes	Girellidae	*Girellastuebeli* Troschel, 1866
Acanthuriformes	Haemulidae	*Parapristipomahumile* (Bowdich ,1825)
	Sparidae	*Diplodusfasciatus* (Valenciennes, 1830)
		*Diploduslineatus* (Valenciennes, 1830)
		*Diplodusprayensis* Cadenat, 1964
		*Virididentexacromegalus* (Osório, 1911)

**Table 2.** List of coastal species from Cabo Verde, with orders and families, following the phylogenetic organisation proposed by Van der Laan et al. (2025).

**Order**	**Family**	**cientific Name**	**References**
Hexanchiformes	Hexanchidae	*Heptranchiasperlo* (Bonnaterre, 1788)	Menezes et al. (2004)
Hexanchiformes	Hexanchidae	*Hexanchusgriseus* (Bonnaterre, 1788)	Hazevoet (2015)
Orectolobiformes	Rhincodontidae	*Rhincodontypus* Smith, 1828	Wirtz et al. (2013), Varela et al. (2025)
Orectolobiformes	Ginglymostomatidae	*Ginglymostomacirratum* (Bonnaterre, 1788)	Wirtz et al. (2013), Freitas et al. (2019a)
Lamniformes	Odontaspididae	*Carchariastaurus* Rafinesque, 1810	Wirtz et al. (2013)
Lamniformes	Odontaspididae	*Odontaspisferox* (Risso, 1810)	Reiner (1996), Menezes et al. (2015)
Lamniformes	Alopiidae	*Alopiassuperciliosus* Lowe, 1841	Wirtz et al. (2013)
Lamniformes	Alopiidae	*Alopiasvulpinus* (Bonnaterre, 1788)	Carpenter and De Angelis (2016a); Eichenbaum (pers. comm. to Rui Freitas)
Lamniformes	Cetorhinidae	*Cetorhinusmaximus* (Gunnerus, 1765)	Wirtz et al. (2013)
Lamniformes	Lamnidae	*Carcharodoncarcharias* (Linnaeus, 1758)	Wirtz et al. (2013)
Lamniformes	Lamnidae	*Isurusoxyrinchus* Rafinesque, 1810	Wirtz et al. (2013)
Lamniformes	Lamnidae	*Isuruspaucus* Guitart Manday, 1966	Wirtz et al. (2013)
Carcharhiniformes	Pseudotriakidae	*Galeorhinusgaleus* (Linnaeus, 1758)	Wirtz et al. (2013)
Carcharhiniformes	Leptochariidae	*Leptochariassmithii* (Müller & Henle, 1839)	Wirtz et al. (2013)
Carcharhiniformes	Triakidae	*Mustelusmustelus* (Linnaeus, 1758)	Krakstad et al. (2011), Menezes et al. (2015)
Carcharhiniformes	Hemigaleidae	*Paragaleuspectoralis* (Garman, 1906)	Wirtz et al. (2013)
Carcharhiniformes	Carcharhinidae	*Carcharhinusaltimus* (Springer, 1950)	Wirtz et al. (2013)
Carcharhiniformes	Carcharhinidae	*Carcharhinusbrevipinna* (Valenciennes, 1839)	Wirtz et al. (2013)
Carcharhiniformes	Carcharhinidae	*Carcharhinusfalciformis* (Bibron, 1839)	Wirtz et al. (2013)
Carcharhiniformes	Carcharhinidae	*Carcharhinusgalapagensis* (Snodgrass & Heller, 1905)	Wirtz et al. (2013)
Carcharhiniformes	Carcharhinidae	*Carcharhinusleucas* (Valenciennes, 1839)	Wirtz et al. (2013)
Carcharhiniformes	Carcharhinidae	*Carcharhinuslimbatus* (Valenciennes, 1839)	Wirtz et al. (2013)
Carcharhiniformes	Carcharhinidae	*Carcharhinuslongimanus* (Poey, 1861)	Wirtz et al. (2013)
Carcharhiniformes	Carcharhinidae	*Carcharhinusobscurus* (Lesueur, 1818)	Wirtz et al. (2013)
Carcharhiniformes	Carcharhinidae	*Carcharhinusplumbeus* (Nardo, 1827)	Wirtz et al. (2013)
Carcharhiniformes	Carcharhinidae	*Carcharhinussignatus* (Poey, 1868)	Wirtz et al. (2013)
Carcharhiniformes	Carcharhinidae	*Galeocerdocuvier* (Péron & Lesueur, 1822)	Wirtz et al. (2013)
Carcharhiniformes	Carcharhinidae	*Negaprionbrevirostris* (Poey, 1868)	Debeius and Kuiter (2006), Wirtz et al. (2013)
Carcharhiniformes	Carcharhinidae	*Prionaceglauca* (Linnaeus, 1758)	Wirtz et al. (2013)
Carcharhiniformes	Carcharhinidae	*Rhizoprionodonacutus* (Rüppell, 1837)	Wirtz et al. (2013)
Carcharhiniformes	Sphyrnidae	*Sphyrnalewini* (Griffith & Smith, 1834)	Varela et al. (2025)
Carcharhiniformes	Sphyrnidae	*Sphyrnamokarran* (Rüppell, 1837)	Wirtz et al. (2013)
Carcharhiniformes	Sphyrnidae	*Sphyrnazygaena* (Linnaeus, 1758)	Wirtz et al. (2013)
Squaliformes	Squalidae	*Squalusmegalops* (Macleay, 1881)	Freitas et al. (2018)
Torpediniformes	Torpedinidae	*Torpedomackayana* Metzelaar, 1919	Ratão et al. (2022)
Torpediniformes	Torpedinidae	*Torpedomarmorata* Risso, 1810	Wirtz et al. (2013)
Rhinopristiformes	Glaucostegidae	*Glaucosteguscemiculus* (Geoffroy St. Hilaire, 1817)	Freitas et al. (2018)
Rajiformes	Rajidae	*Rajaherwigi* Krefft, 1965	Wirtz et al. (2013)
Myliobatiformes	Dasyatidae	*Bathytoshiacentroura* (Mitchill, 1815)	Wirtz et al. (2013)
Myliobatiformes	Dasyatidae	*Dasyatispastinaca* (Linnaeus, 1758)	Krakstad et al. (2011), Wirtz et al. (2013)
Myliobatiformes	Dasyatidae	*Fontitrygonmargarita* (Günther, 1870)	Wirtz et al. (2013)
Myliobatiformes	Dasyatidae	*Pteroplatytrygonviolacea* (Bonaparte, 1832)	Debelius (1997)
Myliobatiformes	Dasyatidae	*Taeniuropsgrabatus* (Geoffroy St.Hilaire, 1817)	Wirtz et al. (2013)
Myliobatiformes	Gymnuridae	*Gymnuraaltavela* (Linnaeus, 1758)	Thomas Eichenbaum, pers. comm. to RF
Myliobatiformes	Aetobatidae	*Aetobatusnarinari* (Euphrasen, 1790)	Debelius (1997), Carpenter and De Angelis (2016a)
Myliobatiformes	Myliobatidae	*Myliobatisaquila* (Linnaeus, 1758)	Wirtz et al. (2013), Carpenter and De Angelis (2016a)
Myliobatiformes	Mobulidae	*Mobulaalfredi* (Krefft, 1868)	Marshall et al. (2009), Wirtz et al. (2013)
Myliobatiformes	Mobulidae	*Mobulabirostris* (Walbaum, 1792)	Wirtz et al. (2013)
Myliobatiformes	Mobulidae	*Mobulatarapacana* (Philippi, 1892)	Wirtz et al. (2013)
Myliobatiformes	Mobulidae	*Mobulathurstoni* (Lloyd, 1908)	Ratão et al. (2017)
Elopiformes	Elopidae	*Elopssenegalensis* Regan, 1909	Freitas et al. (2018)
Elopiformes	Megalopidae	*Megalopsatlanticus* Valenciennes, 1847	Wirtz et al. (2013)
Albuliformes	Albulidae	*Albulagoreensis* Valenciennes, 1847	Quéro et al. (1990)
Albuliformes	Albulidae	*Nemoossisbelloci* (Cadenat, 1937)	Wirtz et al. (2013), Hidaka et al. (2017)
Anguilliformes	Synaphobranchidae	*Synaphobranchusaffinis* Günther, 1877	Almeida et al. (2010), González et al. (2014), González et al. (2021a)
Anguilliformes	Myrocongridae	*Myrocongercompressus* Günther, 1870	González et al. (2014), González et al. (2021)
Anguilliformes	Muraenidae	*Anarchiaslongicauda* (Peter, 1877)	Smith (2012), Wirtz et al. (2013)
Anguilliformes	Muraenidae	*Channomuraenavittata* (Richardson, 1845)	Duarte Lopes et al. (2001), Wirtz et al. (2013)
Anguilliformes	Muraenidae	*Echidnapeli* (Kaup, 1856)	Férnandez-Gil et al. (2013), Wirtz et al. (2013), González et al. (2021b), Mascarenhas (2022)
Anguilliformes	Muraenidae	*Enchelycoreanatina* (Lowe, 1838)	Lipej et al. (2011), Wirtz et al. (2013), González et al. (2021b)
Anguilliformes	Muraenidae	*Enchelycorenigricans* (Bonnaterre, 1788)	Brito et al. (1999), Férnandez-Gil et al. (2013), González et al. (2021b)
Anguilliformes	Muraenidae	*Gymnothoraxafer* Bloch, 1795	Brito et al. (1999), González et al. (2021b)
Anguilliformes	Muraenidae	*Gymnothoraxbacalladoi* Böhlke & Brito, 1987	Brito et al. (1999), González et al. (2021b)
Anguilliformes	Muraenidae	*Gymnothoraxmaderensis* (Johnson, 1862)	González et al. 2014, González et al. 2021b
Anguilliformes	Muraenidae	*Gymnothoraxmiliaris* (Kaup, 1856)	Brito et al. (1999), González et al. (2021b), Mascarenhas (2022)
Anguilliformes	Muraenidae	*Gymnothoraxpolygonius* Poey, 1875	Monteiro (2008), González et al. (2021b)
Anguilliformes	Muraenidae	*Gymnothoraxunicolor* (Delaroche, 1809)	Brito et al. (1999), Férnandez-Gil et al. (2013), González et al. (2021b), Mascarenhas (2022)
Anguilliformes	Muraenidae	*Gymnothoraxvicinus* (Castelnau, 1855)	Monteiro (2008), Férnandez-Gil et al. (2013), González et al. (2021b), Mascarenhas (2022)
Anguilliformes	Muraenidae	*Monopenchelysacuta* (Parr, 1930)	Brito et al. (1999), González et al. (2021b)
Anguilliformes	Muraenidae	*Muraenaaugusti* (Kaup, 1856)	Férnandez-Gil et al. (2013), González et al. (2021b), Mascarenhas (2022)
Anguilliformes	Muraenidae	*Muraenahelena* Linnaeus, 1758	Brito et al. (1999), Férnandez-Gil et al. (2013), González et al. (2021b)
Anguilliformes	Muraenidae	*Muraenamelanotis* (Kaup, 1859)	Brito et al. (1999), Férnandez-Gil et al. (2013), González et al. (2021b), Mascarenhas (2022)
Anguilliformes	Muraenidae	*Muraenarobusta* Osório, 1911	Monteiro (2008), Férnandez-Gil et al. (2013), González et al. (2021b)
Anguilliformes	Muraenidae	*Uropterygiuswheeleri* Blache, 1967	Quéro et al. (1990), Wirtz et al. (2013), González et al. (2021b)
Anguilliformes	Ophichthidae	*Apterichtusanguiformis* (Peters, 1877)	Quéro et al. (1990), Wirtz et al. (2013)
Anguilliformes	Ophichthidae	*Apterichtusmonodi* (Roux, 1966)	Wirtz et al. (2013), Carpenter and De Angelis (2016a)
Anguilliformes	Ophichthidae	*Brachysomophisatlanticus* Blache & Saldanha, 1972	McCosker and Wirtz (2008)
Anguilliformes	Ophichthidae	*Callechelysguineensis* (Osório, 1893)	Wirtz (2022a)
Anguilliformes	Ophichthidae	*Callechelysmuraena* Jordan & Evermann, 1887	Menut and Bérenger (2016), Wirtz (2022b)
Anguilliformes	Ophichthidae	*Echelusmyrus* (Linnaeus, 1758)	Wirtz et al. (2013)
Anguilliformes	Ophichthidae	*Echeluspachyrhynchus* (Vaillant, 1888)	González et al. (2014)
Anguilliformes	Ophichthidae	*Myrichthyspardalis* (Valenciennes, 1839)	González et al. (2014), Mascarenhas (2022)
Anguilliformes	Ophichthidae	*Mystriophisrostellatus* (Richardson, 1848)	Menezes et al. (2004), Wirtz et al. (2013)
Anguilliformes	Ophichthidae	*Ophichthusophis* (Linnaeus, 1758)	D' Oliveira (2010), Menut and Bérenger (2016), Wirtz (2022b)
Anguilliformes	Ophichthidae	*Phaenomonaslongissima* (Cadenat & Marchal, 1963)	Wirtz et al. (2013), González et al. (2014)
Anguilliformes	Nettastomatidae	*Heterocongerlongissimus* Günther, 1870	Wirtz et al. (2013)
Anguilliformes	Congridae	*Ariosomabalearicum* (Delaroche, 1809)	D' Oliveira (2010), Wirtz et al. (2013)
Anguilliformes	Congridae	*Congerconger* (Linnaeus, 1758)	González and Tariche (2008), Wirtz et al. (2013)
Anguilliformes	Congridae	*Gnathophismystax* (Delaroche, 1809)	González et al. (2014)
Anguilliformes	Congridae	*Paracongernotialis* Kanazawa, 1961	Monteiro (2008), Menut and Bérenger (2016)
Clupeiformes	Clupeidae	*Sardinellaaurita* Valenciennes, 1847	Reiner (1996), Wirtz et al. (2013)
Clupeiformes	Clupeidae	*Sardinellamaderensis* (Lowe, 1838)	Monteiro (1998), Wirtz et al. (2013)
Siluriformes	Ariidae	*Carlariuslatiscutatus* (Günther, 1864)	Freitas et al. (2018)
Aulopiformes	Aulopidae	*Aulopusfilamentosus* (Bloch, 1792)	Menezes et al. (2015)
Aulopiformes	Chlorophthalmidae	*Chlorophthalmusagassizi* Bonaparte, 1840	Orrell (2011), Carpenter and De Angelis (2016a)
Aulopiformes	Synodontidae	*Sauridaparri* Norman, 1935	Carpenter and De Angelis (2016a)
Aulopiformes	Synodontidae	*Sauridabrasiliensis* Norman, 1935	Wieber (2011)
Aulopiformes	Synodontidae	*Synodussaurus* (Linnaeus, 1758)	Menezes et al. (2004), Menezes et al. (2015), Menut and Bérenger (2016)
Aulopiformes	Synodontidae	*Synodussynodus* (Linnaeus, 1758)	Menezes et al. (2015), Menut and Bérenger (2016)
Aulopiformes	Synodontidae	*Trachinocephalusmyops* (Forster, 1801)	Quéro et al. (1990)
Lampriformes	Lampridae	*Lamprisguttatus* (Brünnich, 1788)	Carpenter and De Angelis (2016a)
Lampriformes	Trachipteridae	*Zucristatus* (Bonelli, 1819)	Wirtz et al. (2013)
Polymixiiformes	Polymixiidae	*Polymixianobilis* Lowe, 1838	González et al. (2014)
Zeiformes	Zeidae	*Zenopsisconchifer* (Lowe, 1852)	Menezes et al. (2015)
Zeiformes	Zeidae	*Zeusfaber* Linnaeus, 1758	Krakstad et al. (2011), Wirtz et al. (2013)
Gadiformes	Phycidae	*Phycisphycis* (Linnaeus, 1766)	Menezes et al. (2004), Wirtz et al. (2013), Menezes et al. (2015)
Gadiformes	Merlucciidae	*Merlucciussenegalensis* Cadenat, 1950	Wirtz et al. (2013)
Gadiformes	Moridae	*Gadellaimberbis* (Vaillant, 1888)	González et al. (2010), González et al. (2014), Biscoito and Gonzaléz (2017)
Gadiformes	Moridae	*Physiculuscyanostrophus* Anderson & Tweddle, 2002	González et al. (2010), González et al. (2014)
Gadiformes	Moridae	*Physiculusdalwigki* Kaup, 1858	Menezes et al. (2004), González et al. (2010), Menezes et al. (2015)
Gadiformes	Macrouridae	*Coelorinchuscaelorhincus* (Risso, 1810)	Wieber (2011b), Carpenter and De Angelis (2016a)
Gadiformes	Macrouridae	*Nezumiaafricana* (Iwamoto, 1970)	González et al. (2014)
Trachichthyiformes	Trachichthyidae	*Gephyroberyxdarwinii* (Johnson, 1866)	Menezes et al. (2004), González et al. (2014)
Trachichthyiformes	Trachichthyidae	*Hoplostethuscadenati* Quéro, 1974	Reiner (1996), Carpenter and De Angelis (2016a)
Trachichthyiformes	Trachichthyidae	*Hoplostethusmediterraneus* Cuvier, 1829	Reiner (1996), Carpenter and De Angelis (2016a)
Holocentriformes	Holocentridae	*Cornigerspinosus* Agassiz, 1831	Wirtz et al. (2013)
Holocentriformes	Holocentridae	*Myripristisjacobus* Cuvier, 1829	Brito et al. (2006), Menezes et al. (2004), Menezes et al. (2015)
Holocentriformes	Holocentridae	*Sargocentronhastatum* (Cuvier, 1829)	Wirtz et al. (2013)
Ophidiiformes	Ophidiidae	*Brotulabarbata* (Bloch & Schneider, 1801)	Menezes et al. (2004), Wirtz et al. (2013)
Ophidiiformes	Ophidiidae	*Ophidionsaldanhai* Matallanas & Brito, 1999	Matallanas and Brito (1999), Wirtz et al. (2013)
Ophidiiformes	Carapidae	*Carapusacus* (Brünnich, 1768)	Wirtz et al. (2013)
Ophidiiformes	Bythitidae	*Grammonuslonghursti* (Cohen, 1964)	Wirtz (2009)
Batrachoidiformes	Batrachoididae	*Halobatrachusdidactylus* (Bloch & Schneider, 1801)	Reiner (1996), Wirtz et al. (2013)
Gobiiformes	Apogonidae	*Apogonimberbis* (Linnaeus, 1758)	Brito et al. (1999), Freitas et al. (2019a)
Gobiiformes	Apogonidae	*Paroncheilusaffinis* (Poey, 1875)	Brito et al. (1999), Kuiter and Kozawa (2019)
Gobiiformes	Gobiidae	*Bathygobiuscasamancus* (Rochebrune, 1880)	Schliewen (2011), Menut and Bérenger (2016)
Gobiiformes	Gobiidae	*Bathygobiussoporator* (Valenciennes, 1837)	Schliewen (2011), Menut and Bérenger (2016)
Gobiiformes	Gobiidae	*Didogobiuskochi* Van Tassell, 1988	Schliewen (2011), Wirtz et al. (2013)
Gobiiformes	Gobiidae	*Didogobiuswirtzi* Schliewen & Kovačić, 2008	Schliewen and Kovačić (2008), Menut and Bérenger (2016)
Gobiiformes	Gobiidae	*Gnatholepisthompsoni* Jordan, 1904	Larson and Buckle (2012), Menut and Bérenger (2016)
Gobiiformes	Gobiidae	*Gobiusateriformis* Brito & Miller, 2001	Brito and Miller (2001), Menut and Bérenger (2016)
Gobiiformes	Gobiidae	*Gobiussalamansa* Iglésias, Frotté & Sellos, 2015	Iglésias et al. (2015)
Gobiiformes	Gobiidae	*Gobiustetrophthalmus* Brito & Miller, 2001	Brito and Miller (2001), Freitas (2014)
Gobiiformes	Gobiidae	*Marcelogobiusjanetarum* (Schliewen, Wirtz & Kovačić, 2018)	Schliewen et al. (2018)
Gobiiformes	Gobiidae	*Mauligobiusnigri* (Günther, 1861)	Brito and Miller (2001), Schliewen (2011), Freitas (2014)
Gobiiformes	Gobiidae	*Vanneaugobiuscanariensis* Van Tassel, Miller & Brito, 1988	Brito et al. (2006), Schliewen (2011), Wirtz et al. (2013)
Syngnathiformes	Dactylopteridae	*Dactylopterusvolitans* (Linnaeus, 1758)	Brito et al. (1999), Monteiro (2008), Menut and Bérenger (2016)
Syngnathiformes	Mullidae	*Mulloidichthysmartinicus* (Cuvier, 1829)	Brito et al. (1999), Monteiro (2008), Menut and Bérenger (2016)
Syngnathiformes	Mullidae	*Mullusafricanus* Vasil'eva, 2011	Wirtz et al. (2013)
Syngnathiformes	Mullidae	*Pseudupeneusprayensis* (Cuvier, 1829)	Brito et al. (1999), Monteiro (2008), Menut and Bérenger (2016)
Syngnathiformes	Callionymidae	*Callionymusbairdi* Jordan, 1888	Brito et al. (1999), Wirtz et al. (2013), Menut and Bérenger (2016)
Syngnathiformes	Callionymidae	*Synchiropusphaeton* (Günther, 1861)	Krakstad et al. (2011)
Syngnathiformes	Aulostomidae	*Aulostomusstrigosus* Wheeler, 1955	Brito et al. (1999), Menut and Bérenger (2016), Mascarenhas (2022)
Syngnathiformes	Fistulariidae	*Fistulariapetimba* Lacepède, 1803	Brito et al. (1999), Menezes et al. (2004), Wirtz et al. (2013)
Syngnathiformes	Fistulariidae	*Fistulariatabacaria* Linnaeus, 1758	Brito et al. (1999), Menut and Bérenger (2016), Mascarenhas (2022)
Syngnathiformes	Centriscidae	*Macroramphosusscolopax* (Linnaeus, 1758)	Brito et al. (1999), Wirtz et al. (2013)
Syngnathiformes	Syngnathidae	*Hippocampusalgiricus* Kaup, 1856	Wirtz et al. (2013), Menut and Bérenger (2016)
Scombriformes	Ariommatidae	*Ariommamelana* (Ginsburg, 1954)	Krakstad et al. (2011)
Scombriformes	Stromateidae	*Stromateusfiatola* Linnaeus, 1758	Wirtz et al. (2013)
Scombriformes	Pomatomidae	*Pomatomussaltatrix* (Linnaeus, 1766)	Monteiro (1998), Wirtz et al. (2013)
Scombriformes	Scombridae	*Acanthocybiumsolandri* (Cuvier, 1832)	Monteiro (2008), Wirtz et al. (2013)
Scombriformes	Scombridae	*Auxisrochei* (Risso, 1810)	González and Tariche (2008), Wirtz et al. (2013)
Scombriformes	Scombridae	*Auxisthazard* (Lacepède, 1800)	Wirtz et al. (2013), Mascarenhas (2022)
Scombriformes	Scombridae	*Euthynnusalletteratus* (Rafinesque, 1810)	Wirtz et al. (2013), Menut and Bérenger (2016)
Scombriformes	Scombridae	*Katsuwonuspelamis* (Linnaeus, 1758)	Wirtz et al. (2013)
Scombriformes	Scombridae	*Sardasarda* (Bloch, 1793)	Brito et al. (1999), Wirtz et al. (2013)
Scombriformes	Scombridae	*Scombercolias* Gmelin, 1789	Quéro et al. (1990), Brás (2014)
Scombriformes	Scombridae	*Scomberomorustritor* (Cuvier, 1832)	Reiner (1996), Wirtz et al. (2013)
Scombriformes	Scombridae	*Thunnusalalunga* (Bonnaterre, 1788)	Reiner (1996), Wirtz et al. (2013), Carpenter and De Angelis (2016a)
Scombriformes	Scombridae	*Thunnusalbacares* (Bonnaterre, 1788)	Wirtz et al. (2013), Carpenter and De Angelis (2016a)
Scombriformes	Scombridae	*Thunnusobesus* (Lowe, 1839)	González and Tariche (2008), Wirtz et al. (2013), Carpenter and De Angelis (2016a)
Scombriformes	Scombridae	*Thunnusthynnus* (Linnaeus, 1758)	Reiner (1996), Di Natale and Rubio (2013), Carpenter and De Angelis (2016a)
Scombriformes	Gempylidae	*Promethichthysprometheus* (Cuvier, 1832)	González et al. (2014)
Carangiformes	Sphyraenidae	*Sphyraenabarracuda* (Edwards, 1771)	Wirtz et al. (2013)
Carangiformes	Sphyraenidae	*Sphyraenaguachancho* Cuvier, 1829	Wirtz et al. (2013)
Carangiformes	Sphyraenidae	*Sphyraenaviridensis* Cuvier, 1829	Wirtz et al. (2013), Menut and Bérenger (2016)
Carangiformes	Polynemidae	*Galeoidesdecadactylus* (Bloch, 1795)	Brito et al. (1999), Wirtz et al. (2013)
Carangiformes	Citharidae	*Citharuslinguatula* (Linnaeus, 1758)	Reiner (1996), Wirtz et al. (2013)
Carangiformes	Cyclopsettidae	*Citharichthysstampflii* (Steindachner, 1894)	Monteiro (2008), Wirtz et al. (2013)
Carangiformes	Cyclopsettidae	*Syaciumguineense* (Bleeker, 1862)	Brito et al. (1999), Wirtz et al. (2013)
Carangiformes	Bothidae	*Arnoglossusimperialis* (Rafinesque, 1810)	Krakstad et al. (2011)
Carangiformes	Bothidae	*Arnoglossusthori* Kyle, 1913	Quéro et al. (1990), Wirtz et al. (2013)
Carangiformes	Bothidae	*Bothuspodas* (Delaroche, 1809)	Wirtz et al. (2013), Mascarenhas (2022)
Carangiformes	Soleidae	*Microchirushexophthalmus* (Bennett, 1831)	Krakstad et al. (2011)
Carangiformes	Soleidae	*Monochirusatlanticus* Chabanaud, 1940	Wirtz (2017)
Carangiformes	Soleidae	*Pegusacadenati* Chabanaud, 1954	Wirtz et al. (2013), Mascarenhas (2022)
Carangiformes	Cynoglossidae	*Cynoglossuscadenati* Chabanaud, 1947	Fermon et al. (2022)
Carangiformes	Cynoglossidae	*Symphurusinsularis* Munroe, Brito & Hernández, 2000	Munroe et al. (2000)
Carangiformes	Xiphiidae	*Xiphiasgladius* Linnaeus, 1758	Menezes et al. (2004), Carpenter and De Angelis (2016a), González et al. (2020)
Carangiformes	Istiophoridae	*Istiophorusplatypterus* (Shaw, 1792)	Carpenter and De Angelis (2016a)
Carangiformes	Istiophoridae	*Kajikiaalbida* (Poey, 1860)	Carpenter and De Angelis (2016a), González et al. (2020)
Carangiformes	Istiophoridae	*Makairanigricans* Lacepède, 1802	González et al. (2020)
Carangiformes	Istiophoridae	*Tetrapturuspfluegeri* Robins & de Sylva, 1963	González et al. (2020)
Carangiformes	Carangidae	*Alectisciliaris* (Bloch, 1787)	Wirtz et al. (2013), Menut and Bérenger (2016)
Carangiformes	Carangidae	*Caranxcrysos* (Mitchill, 1815)	Menezes et al. (2004), Wirtz et al. (2013), Menut and Bérenger (2016), González et al. (2020), Mascarenhas (2022)
Carangiformes	Carangidae	*Caranxhippos* (Linnaeus, 1766)	Wirtz et al. (2013), Mascarenhas (2022)
Carangiformes	Carangidae	*Caranxlatus* Agassiz, 1831	Wirtz et al. (2013)
Carangiformes	Carangidae	*Caranxlugubris* Poey, 1860	Wirtz et al. (2013), González et al. (2020)
Carangiformes	Carangidae	*Caranxrhonchus* Geoffroy St. Hilaire, 1817	Wirtz et al. (2013)
Carangiformes	Carangidae	*Caranxsenegallus* Cuvier, 1833	Wirtz et al. (2013)
Carangiformes	Carangidae	*Decapterusmacarellus* (Cuvier, 1833)	Wirtz et al. (2013), González et al. (2020)
Carangiformes	Carangidae	*Decapteruspunctatus* (Cuvier, 1829)	Wirtz et al. (2013), González et al. (2020)
Carangiformes	Carangidae	*Decapterustabl* Berry, 1968	González et al. (2020)
Carangiformes	Carangidae	*Elagatisbipinnulata* (Quoy & Gaimard, 1825)	Wirtz et al. (2013), González et al. (2020)
Carangiformes	Carangidae	*Lichiaamia* (Linnaeus, 1758)	Wirtz et al. (2013)
Carangiformes	Carangidae	*Naucratesductor* (Linnaeus, 1758)	Wirtz et al. (2013)
Carangiformes	Carangidae	*Pseudocaranxdentex* (Bloch & Schneider, 1801)	Wirtz et al. (2013), Mascarenhas (2022)
Carangiformes	Carangidae	*Selarcrumenophthalmus* (Bloch, 1793)	Wirtz et al. (2013), González et al. (2020), Mascarenhas (2022)
Carangiformes	Carangidae	*Selenedorsalis* (Gill, 1863)	Wirtz et al. (2013), González et al. (2020), Mascarenhas (2022)
Carangiformes	Carangidae	*Seriolacarpenteri* Mather, 1971	Wirtz et al. (2013)
Carangiformes	Carangidae	*Serioladumerili* (Risso, 1810)	Wirtz et al. (2013)
Carangiformes	Carangidae	*Seriolafasciata* (Bloch, 1793)	Wirtz et al. (2013), González et al. (2020)
Carangiformes	Carangidae	*Seriolarivoliana* Valenciennes, 1833	Wirtz et al. (2013), González et al. (2020), Mascarenhas (2022)
Carangiformes	Carangidae	*Trachinotusgoreensis* Cuvier, 1832	Wirtz et al. (2013), González et al. (2020)
Carangiformes	Carangidae	*Trachinotusmaxillosus* Cuvier, 1832	Carpenter and De Angelis (2016a), Mascarenhas (2022)
Carangiformes	Carangidae	*Trachinotusovatus* (Linnaeus, 1758)	Wirtz et al. (2013), González et al. (2020), Mascarenhas (2022)
Carangiformes	Carangidae	*Trachinotusteraia* Cuvier, 1832	Wirtz et al. (2013)
Carangiformes	Carangidae	*Trachuruspicturatus* (Bowdich, 1825)	Wirtz et al. (2013)
Carangiformes	Carangidae	*Trachurustrecae* Cadenat, 1950	Wirtz et al. (2013), González et al. (2020)
Carangiformes	Carangidae	*Uraspishelvola* (Forster, 1801)	Wirtz et al. (2013), Carpenter and De Angelis (2016a), Brito et al. (2017)
Carangiformes	Echeneidae	*Echeneisnaucrates* Linnaeus, 1758	Wirtz et al. (2013)
Carangiformes	Echeneidae	*Remorabrachyptera* (Lowe, 1839)	Wirtz et al. (2013)
Carangiformes	Echeneidae	*Remoraremora* (Linnaeus, 1758)	Wirtz et al. (2013)
Carangiformes	Rachycentridae	*Rachycentroncanadum* (Linnaeus, 1766)	Freitas et al. (2018)
Carangiformes	Coryphaenidae	*Coryphaenaequiselis* Linnaeus, 1758	Wirtz et al. (2013)
Carangiformes	Coryphaenidae	*Coryphaenahippurus* Linnaeus, 1758	Wirtz et al. (2013), González et al. (2020)
Atheriniformes	Atherinidae	*Atherinalopeziana* Rossignol & Blache, 1961	Wirtz et al. (2013), Mascarenhas (2022)
Beloniformes	Scomberesocidae	*Scomberesoxsaurus* (Walbaum, 1792)	Wirtz et al. (2013)
Beloniformes	Belonidae	*Ablenneshians* (Valenciennes, 1846)	Wirtz et al. (2013), González et al. (2020)
Beloniformes	Belonidae	*Beloneacus* Risso, 1827	Wirtz et al. (2013)
Beloniformes	Belonidae	*Platybelonelovii* (Günther, 1866)	Wirtz et al. (2013), Mascarenhas (2022)
Beloniformes	Belonidae	*Tylosuruscrocodilus* (Péron & Lesueur, 1821	Wirtz et al. (2013)
Beloniformes	Belonidae	*Tylosurusimperialis* (Rafinesque, 1810)	Wirtz et al. (2013)
Beloniformes	Belonidae	*Tylosurusrafale* Collette & Parin, 1970	Wirtz et al. (2013), González et al. (2020)
Beloniformes	Hemiramphidae	*Euleptorhamphusvelox* Poey, 1868	Wirtz et al. (2013)
Beloniformes	Hemiramphidae	*Hemiramphusbalao* Lesueur, 1821	Wirtz et al. (2013)
Beloniformes	Hemiramphidae	*Hemiramphusbrasiliensis* (Linnaeus, 1758)	Wirtz et al. (2013)
Beloniformes	Hemiramphidae	*Oxyporhamphussimilis* Bruun, 1935	Wirtz et al. (2013)
Beloniformes	Exocoetidae	*Cheilopogoncyanopterus* (Valenciennes, 1847)	Wirtz et al. (2013)
Beloniformes	Exocoetidae	*Cheilopogonexsiliens* (Linnaeus, 1771)	Dooley et al. (2015)
Beloniformes	Exocoetidae	*Cheilopogonfurcatus* (Mitchill, 1815)	Wirtz et al. (2013), Carpenter and De Angelis (2016a)
Beloniformes	Exocoetidae	*Cheilopogonpinnatibarbatus* (Bennett, 1831)	Wirtz et al. (2013), Carpenter and De Angelis (2016a)
Beloniformes	Exocoetidae	*Exocoetusobtusirostris* Günther, 1866	Wirtz et al. (2013), Carpenter and De Angelis (2016a)
Beloniformes	Exocoetidae	*Exocoetusvolitans* Linnaeus, 1758	Wirtz et al. (2013), Carpenter and De Angelis (2016a)
Beloniformes	Exocoetidae	*Prognichthysgibbifrons* (Valenciennes, 1847)	Wirtz et al. (2013)
Cichliformes	Pomacentridae	*Abudefdufhoefleri* (Steindachner, 1881)	Wirtz et al. (2013), Carpenter and De Angelis (2016b), Mascarenhas (2022)
Cichliformes	Pomacentridae	*Abudefdufsaxatilis* (Linnaeus, 1758)	Wirtz et al. (2013), Carpenter and De Angelis (2016b), Mascarenhas (2022)
Cichliformes	Pomacentridae	*Abudefduftaurus* (Müller & Troschel, 1848)	Wirtz et al. (2013), Carpenter and De Angelis (2016b), Mascarenhas (2022)
Cichliformes	Pomacentridae	*Azurinamultilineata* (Guichenot, 1853)	Wirtz et al. (2013), Carpenter and De Angelis (2016b), Mascarenhas (2022)
Cichliformes	Pomacentridae	*Chromislubbocki* Edwards, 1986	Wirtz et al. (2013), Carpenter and De Angelis (2016b), Mascarenhas (2022)
Cichliformes	Pomacentridae	*Similiparmahermani* (Steindachner, 1887)	Wirtz et al. (2013), Carpenter and De Angelis (2016b), Mascarenhas (2022)
Cichliformes	Pomacentridae	*Similiparmalurida* (Cuvier, 1830)	Wirtz et al. (2013), Carpenter and De Angelis (2016b)
Cichliformes	Pomacentridae	*Stegastesimbricatus* Jenyns, 1840	Wirtz et al. (2013), Carpenter and De Angelis (2016b), Mascarenhas (2022)
Mugiliformes	Mugilidae	*Chelonbispinosus* (Bowdich, 1825)	Wirtz et al. (2013), Carpenter and De Angelis (2016a), Mascarenhas (2022)
Mugiliformes	Mugilidae	*Chelonlabrosus* (Risso, 1827)	Wirtz et al. (2013), Carpenter and De Angelis (2016a)
Mugiliformes	Mugilidae	*Mugilbananensis* (Pellegrin, 1927)	Wirtz et al. (2013), Mascarenhas (2022)
Mugiliformes	Mugilidae	*Mugilcapurrii* (Perugia, 1892)	Wirtz et al. (2013)
Mugiliformes	Mugilidae	*Mugilcephalus* Linnaeus, 1758	Freitas et al. (2018)
Mugiliformes	Mugilidae	*Mugilcurema* Valenciennes, 1836	Wirtz et al. (2013)
Blenniiformes	Gobiesocidae	*Apletodonbarbatus* Fricke, Wirtz & Brito, 2010	Wirtz et al. (2013)
Blenniiformes	Gobiesocidae	*Diplecogasterpectoralis* Briggs, 1955	Wirtz et al. (2013)
Blenniiformes	Blenniidae	*Entomacroduscadenati* Springer, 1967	Wirtz et al. (2013), Menut and Bérenger (2016)
Blenniiformes	Blenniidae	*Microlipophryscaboverdensis* (Wirtz & Bath, 1989)	Wirtz et al. (2013), Menut and Bérenger (2016), Mascarenhas (2022)
Blenniiformes	Blenniidae	*Ophioblenniusatlanticus* (Valenciennes, 1836)	Wirtz et al. (2013), Menut and Bérenger (2016), Mascarenhas (2022)
Blenniiformes	Blenniidae	*Parablenniusparvicornis* (Valenciennes, 1836)	Wirtz et al. (2013), Menut and Bérenger (2016), Mascarenhas (2022)
Blenniiformes	Blenniidae	*Parablenniussalensis* Bath, 1990	Wirtz et al. (2013), Menut and Bérenger (2016), Mascarenhas (2022)
Blenniiformes	Blenniidae	*Scartellacaboverdiana* Bath, 1990	Wirtz et al. (2013), Menut and Bérenger (2016), Mascarenhas (2022)
Blenniiformes	Labrisomidae	*Labrisomusnuchipinnis* (Quoy & Gaimard, 1824)	Wirtz et al. (2013), Mascarenhas (2022)
Blenniiformes	Labrisomidae	*Malacoctenuscarrowi* Wirtz, 2014	Wirtz (2014), Mascarenhas (2022)
Perciformes	Serranidae	*Anthiasanthias* (Linnaeus, 1758)	Wirtz et al. (2013), González et al. (2014)
Perciformes	Serranidae	*Mycteropercafusca* (Lowe, 1838)	Wirtz et al. (2013), Menut and Bérenger (2016), González et al. (2020), Mascarenhas (2022)
Perciformes	Serranidae	*Serranusatricauda* Günther, 1874	Wirtz et al. (2013), Menut and Bérenger (2016), González et al. (2020)
Perciformes	Serranidae	*Serranuscabrilla* (Linnaeus, 1758)	Freitas et al. (2018)
Perciformes	Serranidae	*Serranusheterurus* (Cadenat, 1937)	Wirtz et al. 2013, Freitas et al. 2018
Perciformes	Epinephelidae	*Cephalopholistaeniops* (Valenciennes, 1828)	Wirtz et al. (2013), Menut and Bérenger (2016), González et al. (2020), Mascarenhas (2022)
Perciformes	Epinephelidae	*Epinepheluscostae* (Steindachner, 1878)	Wirtz et al. (2013)
Perciformes	Epinephelidae	*Epinephelusgoreensis* (Valenciennes, 1830)	Wirtz et al. (2013), González et al. (2014), González et al. (2020)
Perciformes	Epinephelidae	*Epinephelusmarginatus* (Lowe, 1834)	Wirtz et al. (2013), Menut and Bérenger (2016), Freitas et al. (2019a), Mascarenhas (2022)
Perciformes	Liopropomatidae	*Liopropomaemanueli* Wirtz & Schliewen, 2012	Wirtz et al. (2013), Freitas (2014), Menut and Bérenger (2016)
Perciformes	Grammistidae	*Pseudogrammaguineensis* (Norman, 1935)	Wirtz et al. (2013), Menut and Bérenger (2016)
Perciformes	Grammistidae	*Rypticussaponaceus* (Bloch & Schneider, 1801)	Wirtz et al. (2013), Menut and Bérenger (2016), Freitas et al. (2019a)
Perciformes	Trachinidae	*Trachinusarmatus* Bleeker, 1861	Wirtz et al. (2013), Carpenter and De Angelis (2016b)
Perciformes	Trachinidae	*Trachinuspellegrini* Cadenat, 1937	Wirtz et al. (2013), Carpenter and De Angelis (2016b)
Perciformes	Triglidae	*Chelidonichthysgabonensis* (Poll & Roux, 1955)	Krakstad et al. (2011), Wirtz et al. (2013)
Perciformes	Triglidae	*Chelidonichthyslastoviza* (Bonnaterre, 1788)	Krakstad et al. (2011)
Perciformes	Triglidae	*Lepidotriglacadmani* Regan, 1915	Wirtz et al. (2013)
Perciformes	Scorpaenidae	*Helicolenusdactylopterus* (Delaroche, 1809)	González et al. (2014), Menezes et al. (2015), González et al. (2020)
Perciformes	Scorpaenidae	*Pontinusaccraensis* Norman, 1935	Menezes et al. (2015)
Perciformes	Scorpaenidae	*Pontinuskuhlii* (Bowdich, 1825)	González et al. (2014), Menezes et al. (2015), González et al. (2020)
Perciformes	Scorpaenidae	*Scorpaenaangolensis* Norman, 1935	Wirtz et al. (2013), Menezes et al. (2015), Carpenter and De Angelis (2016a)
Perciformes	Scorpaenidae	*Scorpaenaelongata* Cadenat, 1943	González et al. (2014), Menezes et al. (2015), Carpenter and De Angelis (2016a)
Perciformes	Scorpaenidae	*Scorpaenalaevis* Troschel, 1866	Wirtz et al. (2013), González et al. (2014), Carpenter and De Angelis (2016a), Mascarenhas (2022)
Perciformes	Scorpaenidae	*Scorpaenamaderensis* Valenciennes, 1833	Wirtz et al. (2013), Carpenter and De Angelis (2016a), Mascarenhas (2022)
Perciformes	Scorpaenidae	*Scorpaenanotata* Rafinesque, 1810	Wirtz et al. (2013), Carpenter and De Angelis (2016a)
Perciformes	Scorpaenidae	*Scorpaenascrofa* Linnaeus, 1758	Wirtz et al. (2013), Menezes et al. (2015), Carpenter and De Angelis (2016a), González et al. (2020)
Perciformes	Scorpaenidae	*Scorpaenastephanica* Cadenat, 1943	Wirtz et al. (2013), Russell et al. (2015)
Perciformes	Scorpaenidae	*Setarchesguentheri* Johnson, 1862	Carpenter and De Angelis (2016a)
Labriformes	Labridae	*Acantholabruspalloni* (Risso, 1810)	Wirtz et al. (2013), Carpenter and De Angelis (2016b), González et al. (2018)
Labriformes	Labridae	*Bodianusscrofa* (Valenciennes, 1839)	Wirtz et al. (2013), Menezes et al. (2015), González et al. (2020)
Labriformes	Labridae	*Bodianusspeciosus* (Bowdich, 1825)	Wirtz et al. (2013), Menut and Bérenger (2016), Freitas et al. (2019a)
Labriformes	Labridae	*Corisatlantica* Günther, 1862	Wirtz et al. (2013), Menut and Bérenger (2016), Freitas et al. (2019a), Mascarenhas (2022)
Labriformes	Labridae	*Doratonotusmegalepis* Günther, 1862	Wirtz et al. (2013), Carpenter and De Angelis (2016b)
Labriformes	Labridae	*Lappanellafasciata* (Cocco, 1833)	González et al. (2014)
Labriformes	Labridae	*Scarushoefleri* (Steindachner, 1881)	Wirtz et al. (2013), Freitas et al. (2019a), Mascarenhas (2022)
Labriformes	Labridae	*Sparisomachoati* Rocha, Brito & Robertson, 2012	Wirtz et al. (2013), Freitas et al. (2019a), Mascarenhas (2022)
Labriformes	Labridae	*Sparisomacretense* (Linnaeus, 1758)	Wirtz et al. (2013), Freitas et al. (2019a), Mascarenhas (2022)
Labriformes	Labridae	*Sparisomafrondosum* (Agassiz, 1831)	Freitas et al. (2014), Menut and Bérenger (2016)
Labriformes	Labridae	*Thalassomanewtoni* (Osório, 1891)	Reiner (2005) Fig. 7
Labriformes	Labridae	*Thalassomapavo* (Linnaeus, 1758)	Wirtz et al. (2013), Menut and Bérenger (2016), Freitas et al. (2019a), Mascarenhas (2022)
Labriformes	Labridae	*Xyrichtysnovacula* (Linnaeus, 1758)	Wirtz et al. (2013), Carpenter and De Angelis (2016b), Menut and Bérenger (2016), Mascarenhas (2022)
Labriformes	Uranoscopidae	*Uranoscopuscadenati* Poll, 1959	Wirtz et al. (2013), Menut and Bérenger (2016)
Labriformes	Uranoscopidae	*Uranoscopuspolli* Cadenat, 1951	Reiner (1996), Krakstad et al. (2011)
Labriformes	Pinguipedidae	*Parapercisatlantica* (Vaillant, 1887)	Krakstad et al. (2011), Carpenter and De Angelis (2016b)
Centrarchiformes	Girellidae	*Girellastuebeli* Troschel, 1866	Wirtz et al. (2013), Freitas (2014), Menut and Bérenger (2016), Mascarenhas (2022)
Centrarchiformes	Kyphosidae	*Kyphosusbigibbus* Lacepède, 1801	Carpenter and De Angelis (2016b), Knudsen and Clements (2016)
Centrarchiformes	Kyphosidae	*Kyphosussectatrix* (Linnaeus, 1758)	Knudsen and Clements (2013), Knudsen and Clements (2016)
Centrarchiformes	Kyphosidae	*Kyphosusvaigiensis* (Quoy & Gaimard, 1825)	Bañon and Carlos (2022)
Acropomatiformes	Epigonidae	*Epigonusconstanciae* (Giglioli, 1880)	Wirtz et al. (2013)
Acropomatiformes	Polyprionidae	*Polyprionamericanus* (Bloch & Schneider, 1801)	Wirtz et al. (2013)
Acanthuriformes	Gerreidae	*Eucinostomusmelanopterus* (Bleeker, 1863)	Wirtz et al. (2013), Menut and Bérenger (2016), Mascarenhas (2022)
Acanthuriformes	Gerreidae	*Gerresnigri* Günther, 1859	Wirtz et al. (2013)
Acanthuriformes	Ephippidae	*Chaetodipteruslippei* Steindachner, 1895	Wirtz et al. (2013), Carpenter and De Angelis (2016b)
Acanthuriformes	Ephippidae	*Ephippusgoreensis* Cuvier, 1831	Wirtz et al. (2013), Carpenter and De Angelis (2016b), Menut and Bérenger (2016)
Acanthuriformes	Sciaenidae	*Sciaenaumbra* Linnaeus, 1758	Wirtz et al. (2013), Mascarenhas (2022)
Acanthuriformes	Sciaenidae	*Umbrinaronchus* Valenciennes, 1843	Wirtz et al. (2013), Freitas et al. (2019a)
Acanthuriformes	Haemulidae	*Parapristipomahumile* (Bowdich, 1825)	Wirtz et al. (2013), Freitas et al. (2019a), Mascarenhas (2022)
Acanthuriformes	Haemulidae	*Parapristipomamacrops* (Pellegrin, 1912)	González and Tariche (2008)
Acanthuriformes	Haemulidae	*Parapristipomaoctolineatum* (Valenciennes, 1833)	Wirtz et al. (2013), Carpenter and De Angelis (2016b), Freitas et al. (2019a)
Acanthuriformes	Haemulidae	*Pomadasysincisus* (Bowdich, 1825)	Wirtz et al. (2013), Krakstad et al. (2011), Menezes et al. (2015)
Acanthuriformes	Haemulidae	*Pomadasysjubelini* (Cuvier, 1830)	Mascarenhas (2022)
Acanthuriformes	Haemulidae	*Pomadasysperotaei* (Cuvier, 1830)	Wirtz et al. (2013)
Acanthuriformes	Haemulidae	*Pomadasysrogerii* (Cuvier, 1830)	Wirtz et al. (2013), Menut and Bérenger (2016)
Acanthuriformes	Lobotidae	*Lobotessurinamensis* (Bloch, 1790)	Wirtz et al. (2013)
Acanthuriformes	Emmelichthyidae	*Erythroclesmonodi* Poll & Cadenat, 1954	Menezes et al. (2015)
Acanthuriformes	Lutjanidae	*Apsilusfuscus* Valenciennes, 1830	Wirtz et al. (2013), González et al. (2020)
Acanthuriformes	Lutjanidae	*Lutjanusagennes* Bleeker, 1863	Wirtz et al. (2013), Menezes et al. (2015)
Acanthuriformes	Lutjanidae	*Lutjanusdentatus* (Duméril, 1861)	Freitas et al. (2018)
Acanthuriformes	Lutjanidae	*Lutjanusfulgens* (Valenciennes, 1830)	Wirtz et al. (2013), Freitas et al. (2018), González et al. (2020)
Acanthuriformes	Lutjanidae	*Lutjanusgoreensis* (Valenciennes, 1830)	Wirtz et al. (2013), Freitas et al. (2018)
Acanthuriformes	Latilidae	*Branchiostegussemifasciatus* (Norman, 1931)	Freitas et al. (2018)
Acanthuriformes	Pomacanthidae	*Holacanthusafricanus* Cadenat, 1951	Wirtz et al. 2013, Menut and Bérenger 2016, Freitas et al. 2019a
Acanthuriformes	Chaetodontidae	*Chaetodonhoefleri* Steindachner, 1881	Wirtz et al. (2013), Carpenter and De Angelis (2016b)
Acanthuriformes	Chaetodontidae	*Chaetodonrobustus* Günther, 1860	Wirtz et al. (2013), Carpenter and De Angelis (2016b), Mascarenhas (2022)
Acanthuriformes	Chaetodontidae	*Prognathodesmarcellae* (Poll, 1950)	Wirtz et al. (2013), Carpenter and De Angelis (2016b), Menut and Bérenger (2016)
Acanthuriformes	Luvaridae	*Luvarusimperialis* Rafinesque, 1810	Reiner (1996)
Acanthuriformes	Acanthuridae	*Acanthuruschirurgus* (Bloch, 1787)	Brito et al. (1999), Wirtz et al. (2013)
Acanthuriformes	Acanthuridae	*Acanthurusmonroviae* Steindachner, 1876	Wirtz et al. (2013), Menut and Bérenger (2016), Freitas et al. (2019a), Mascarenhas (2022)
Acanthuriformes	Lethrinidae	*Lethrinusatlanticus* Valenciennes, 1830	Wirtz et al. (2013), Menut and Bérenger (2016), González et al. (2020)
Acanthuriformes	Sparidae	*Boopsboops* (Linnaeus, 1758)	Wirtz et al. (2013), González et al. (2020), Mascarenhas (2022)
Acanthuriformes	Sparidae	*Dentexmacrophthalmus* (Bloch, 1791)	Wirtz et al. (2013), Menezes et al. (2015)
Acanthuriformes	Sparidae	*Diplodusfasciatus* (Valenciennes, 1830)	Wirtz et al. (2013), Menut and Bérenger (2016), Mascarenhas (2022)
Acanthuriformes	Sparidae	*Diploduslineatus* (Valenciennes, 1830)	Freitas et al. (2019a), González et al. (2020), Mascarenhas (2022)
Acanthuriformes	Sparidae	*Diplodusprayensis* Cadenat, 1964	Wirtz et al. (2013), Menut and Bérenger (2016), Mascarenhas (2022)
Acanthuriformes	Sparidae	*Diploduspuntazzo* (Walbaum, 1792)	Wirtz et al. (2013)
Acanthuriformes	Sparidae	*Lithognathusmormyrus* (Linnaeus, 1758)	Wirtz et al. (2013), Menezes et al. (2015), Menut and Bérenger (2016)
Acanthuriformes	Sparidae	*Obladamelanurus* (Linnaeus, 1758)	Wirtz et al. (2013), Morri et al. (2000)
Acanthuriformes	Sparidae	*Pagellusacarne* (Risso, 1827)	Wirtz et al. (2013), Menezes et al. (2015), González et al. (2020)
Acanthuriformes	Sparidae	*Pagelluserythrinus* (Linnaeus, 1758)	Wirtz et al. (2013), Carpenter and De Angelis (2016b)
Acanthuriformes	Sparidae	*Pagrusafricanus* Akazaki, 1962	Wirtz et al. (2013), Menezes et al. (2015), González et al. (2020)
Acanthuriformes	Sparidae	*Pagrusauriga* Valenciennes, 1843	Wirtz et al. (2013), Freitas et al. (2018)
Acanthuriformes	Sparidae	*Sarpasalpa* (Linnaeus, 1758)	Wirtz et al. (2013), Carpenter and De Angelis (2016b), Mascarenhas (2022)
Acanthuriformes	Sparidae	*Spicaramelanurus* (Valenciennes, 1830)	Wirtz et al. (2013), Freitas et al. (2019a), Mascarenhas (2022)
Acanthuriformes	Sparidae	*Spondyliosomacantharus* (Linnaeus, 1758)	Wirtz et al. (2013), Menezes et al. (2015), González et al. (2020)
Acanthuriformes	Sparidae	*Virididentexacromegalus* (Osório, 1911)	Wirtz et al. (2013), Menezes et al. (2015), González et al. (2020), Mascarenhas (2022)
Acanthuriformes	Cepolidae	*Cepolapauciradiata* Cadenat, 1950	Wirtz et al. (2013)
Acanthuriformes	Priacanthidae	*Heteropriacanthuscruentatus* (Lacepède, 1801)	Wirtz et al. (2013), Menut and Bérenger (2016), Mascarenhas (2022)
Acanthuriformes	Priacanthidae	*Heteropriacanthusfulgens* (Lowe, 1838)	Fernandez-Silva and Ho (2017), Freitas et al. (2019a)
Acanthuriformes	Priacanthidae	*Priacanthusarenatus* Cuvier, 1829	Wirtz et al. (2013), González et al. (2020), Mascarenhas (2022)
Acanthuriformes	Caproidae	*Antigoniacapros* Lowe, 1843	Krakstad et al. (2011), González et al. (2014)
Acanthuriformes	Caproidae	*Caprosaper* (Linnaeus, 1758)	Wirtz et al. (2013), González et al. (2014), Carpenter and De Angelis (2016b)
Lophiiformes	Lophiidae	*Lophiusvaillanti* Regan, 1903	Quéro et al. (1990), Menezes et al. (2015), Carpenter and De Angelis (2016a), Freitas et al. (2018)
Lophiiformes	Antennariidae	*Abantennariusnummifer* (Cuvier, 1817)	D' Oliveira (2010), Wirtz et al. (2013), Wirtz (2022c)
Lophiiformes	Antennariidae	*Antennariusmultiocellatus* (Valenciennes, 1837)	Wirtz (2022c)
Lophiiformes	Antennariidae	*Antennariuspardalis* (Valenciennes, 1837)	Mascarenhas (2022), Wirtz (2022c)
Lophiiformes	Antennariidae	*Antennariusstriatus* (Shaw, 1794)	Wirtz et al. (2013), Mascarenhas (2022), Wirtz (2022c)
Lophiiformes	Antennariidae	*Fowlerichthyssenegalensis* Cadenat, 1959	Wirtz et al. (2013), Menut and Bérenger (2016), Mascarenhas (2022), Wirtz (2022c)
Lophiiformes	Antennariidae	*Histriohistrio* (Linnaeus, 1758)	Menut and Bérenger (2016), Wirtz et al. (2016), Wirtz (2022c)
Tetraodontiformes	Molidae	*Masturuslanceolatus* (Liénard, 1840)	Wirtz et al. (2013)
Tetraodontiformes	Molidae	*Molaalexandrini* (Ranzani, 1834)	D' Oliveira (2010), Wirtz and Biscoito (2019)
Tetraodontiformes	Molidae	*Ranzanialaevis* (Pennant, 1776)	Wirtz et al. (2013)
Tetraodontiformes	Diodontidae	*Chilomycterusmauretanicus* (Le Danois, 1954)	Wirtz et al. (2013), Mascarenhas (2022)
Tetraodontiformes	Diodontidae	*Chilomycterusreticulatus* (Linnaeus, 1758)	Wirtz et al. (2013), Menezes et al. (2015), Menut and Bérenger (2016), Mascarenhas (2022)
Tetraodontiformes	Diodontidae	*Diodoneydouxii* Brisout de Barneville, 1846	Rui Freitas person. obser.; Fig. 8
Tetraodontiformes	Diodontidae	*Diodonholocanthus* Linnaeus, 1758	Wirtz et al. (2013), Menut and Bérenger (2016), Freitas et al. (2019b), Mascarenhas (2022)
Tetraodontiformes	Diodontidae	*Diodonhystrix* Linnaeus, 1758	Wirtz et al. (2013), Menut and Bérenger (2016), Freitas et al. (2019b), Mascarenhas (2022)
Tetraodontiformes	Tetraodontidae	*Canthigastercapistrata* (Lowe, 1839)	Wirtz et al. (2013), Menut and Bérenger (2016), Freitas et al. (2019a), Mascarenhas (2022)
Tetraodontiformes	Tetraodontidae	*Canthigastersupramacula* Moura & Castro, 2002	Wirtz et al. (2013)
Tetraodontiformes	Tetraodontidae	*Lagocephaluslagocephalus* (Linnaeus, 1758)	Wirtz et al. (2013), Menezes et al. (2015)
Tetraodontiformes	Tetraodontidae	*Sphoeroidesmarmoratus* (Lowe, 1838)	Wirtz et al. (2013), Menut and Bérenger (2016), Freitas et al. (2019a), Mascarenhas (2022)
Tetraodontiformes	Tetraodontidae	*Sphoeroidespachygaster* (Müller & Troschel, 1848)	Wirtz et al. (2013), Menezes et al. (2015)
Tetraodontiformes	Monacanthidae	*Aluterusheudelotii* Hollard, 1855	Wirtz et al. (2013), Krakstad et al. (2011), Menut and Bérenger (2016), Mascarenhas (2022)
Tetraodontiformes	Monacanthidae	*Aluterusmonoceros* (Linnaeus, 1758)	Krakstad et al. (2011)
Tetraodontiformes	Monacanthidae	*Aluterusscriptus* (Osbeck, 1765)	Wirtz et al. (2013), Krakstad et al. (2011), Menut and Bérenger (2016)
Tetraodontiformes	Monacanthidae	*Aluterusschoepfii* (Walbaum, 1792)	Wirtz et al. (2013), Krakstad et al. (2011)
Tetraodontiformes	Monacanthidae	*Cantherhinesmacrocerus* (Hollard, 1853)	Wirtz et al. (2013)
Tetraodontiformes	Monacanthidae	*Stephanolepishispida* (Linnaeus, 1766)	Krakstad et al. (2011), Wirtz et al. (2013), Menut and Bérenger (2016), Mascarenhas (2022)
Tetraodontiformes	Balistidae	*Balistescapriscus* Gmelin, 1789	Wirtz et al. (2013), Menezes et al. (2015)
Tetraodontiformes	Balistidae	*Balistespunctatus* Gmelin, 1789	Wirtz et al. (2013), Freitas et al. (2019a), Mascarenhas (2022)
Tetraodontiformes	Balistidae	*Balistesvetula* Linnaeus, 1758	Wirtz et al. (2013)
Tetraodontiformes	Balistidae	*Canthidermismaculata* (Bloch, 1786)	Wirtz et al. (2013)
Tetraodontiformes	Balistidae	*Canthidermissufflamen* (Mitchill, 1815)	Wirtz et al. (2013), Menezes et al. (2015), Freitas et al. (2019a)
Tetraodontiformes	Balistidae	*Melichthysniger* (Bloch, 1786)	Wirtz et al. (2013)
Tetraodontiformes	Balistidae	*Rhinecanthusaculeatus* (Linnaeus, 1758)	Wirtz et al. (2013)

**Table 3.** Higher taxomomic levels (class, order and family) with the number of families by order and species by family.

**Class**	**Order**	**Family**	**Family by Order**	**Species by Family**
Elasmobranchii				
	Hexanchiformes		1	
		Hexanchidae		2
	Orectolobiformes		2	
		Rhincodontidae		1
		Ginglymostomatidae		1
	Lamniformes		4	
		Odontaspididae		2
		Alopiidae		2
		Cetorhinidae		1
		Lamnidae		3
	Carcharhiniformes		6	
		Pseudotriakidae		1
		Leptochariidae		1
		Triakidae		1
		Hemigaleidae		1
		Carcharhinidae		14
		Sphyrnidae		3
	Squaliformes		1	
		Squalidae		1
	Torpediniformes		1	
		Torpedinidae		2
	Rhinopristiformes		1	
		Glaucostegidae		1
	Rajiformes		1	
		Rajidae		1
	Myliobatiformes		5	
		Dasyatidae		5
		Gymnuridae		1
		Aetobatidae		1
		Myliobatidae		1
		Mobulidae		4
Actinopteri				
	Elopiformes		2	
		Elopidae		1
		Megalopidae		1
	Albuliformes		1	
		Albulidae		2
	Anguilliformes		6	
		Synaphobranchidae		1
		Myrocongridae		1
		Muraenidae		18
		Ophichthidae		11
		Nettastomatidae		1
		Congridae		4
	Clupeiformes		1	
		Clupeidae		2
	Siluriformes		1	
		Ariidae		1
	Aulopiformes		3	
		Aulopidae		1
		Chlorophthalmidae		1
		Synodontidae		5
	Lampriformes		2	
		Lampridae		1
		Trachipteridae		1
	Polymixiiformes		1	
		Polymixiidae		1
	Zeiformes		1	
		Zeidae		2
	Gadiformes		4	
		Phycidae		1
		Merlucciidae		1
		Moridae		3
		Macrouridae		2
	Trachichthyiformes		1	
		Trachichthyidae		3
	Beryciformes		1	
		Holocentridae		3
	Ophidiiformes		3	
		Ophidiidae		2
		Carapidae		1
		Bythitidae		1
	Batrachoidiformes		1	
		Batrachoididae		1
	Gobiiformes		2	
		Apogonidae		2
		Gobiidae		11
	Syngnathiformes		7	
		Dactylopteridae		1
		Mullidae		3
		Callionymidae		2
		Aulostomidae		1
		Fistulariidae		2
		Centriscidae		1
		Syngnathidae		1
	Scombriformes		5	
		Ariommatidae		1
		Stromateidae		1
		Pomatomidae		1
		Scombridae		12
		Gempylidae		1
	Carangiformes		13	
		Sphyraenidae		3
		Polynemidae		1
		Citharidae		1
		Cyclopsettidae		2
		Bothidae		3
		Soleidae		3
		Cynoglossidae		2
		Xiphiidae		1
		Istiophoridae		4
		Carangidae		27
		Echeneidae		3
		Rachycentridae		1
		Coryphaenidae		2
	Atheriniformes		1	
		Atherinidae		1
	Beloniformes		4	
		Scomberesocidae		1
		Belonidae		6
		Hemiramphidae		4
		Exocoetidae		7
	Cichliformes		1	
		Pomacentridae		8
	Mugiliformes		1	
		Mugilidae		6
	Blenniiformes		3	
		Gobiesocidae		2
		Blenniidae		6
		Labrisomidae		2
	Perciformes		10	
		Serranidae		5
		Epinephelidae		4
		Liopropomatidae		1
		Grammistidae		2
		Trachinidae		2
		Triglidae		3
		Scorpaenidae		11
		Labridae		13
		Uranoscopidae		2
		Pinguipedidae		1
	Centrarchiformes		2	
		Girellidae		1
		Kyphosidae		3
	Acropomatiformes		2	
		Epigonidae		1
		Polyprionidae		1
	Acanthuriformes		17	
		Gerreidae		2
		Ephippidae		2
		Sciaenidae		2
		Haemulidae		7
		Lobotidae		1
		Emmelichthyidae		1
		Lutjanidae		5
		Latilidae		1
		Pomacanthidae		1
		Chaetodontidae		3
		Luvaridae		1
		Acanthuridae		2
		Lethrinidae		1
		Sparidae		16
		Cepolidae		1
		Priacanthidae		3
		Caproidae		2
	Lophiiformes		2	
		Lophiidae		1
		Antennariidae		6
	Tetraodontiformes		5	
		Molidae		3
		Diodontidae		5
		Tetraodontidae		5
		Monacanthidae		6
		Balistidae		7

**Table 4.** Taxonomic levels with numbers.

**Taxon**	**number**
Order	39
Family	125
Genus	266
Species	393

**Table 5.** Unconfirmed occurrences not inclued on the main checklist.

Family	Species	Occurrence status	Reference	Observations
Torpedinidae	*Torpedotorpedo* (Linnaeus, 1758)	Need Confirmation	Reiner (2005)	The photo in Reiner (2005) shows *Torpedomarmorata* Risso, 1810
Atherinidae	*Atherinapresbyter* Cuvier, 1829	Doubtful	Mugé in Quéro et al. (1990)	Probably a confusion with *Atherinalopeziana* Rossignol & Blache 1961
Scorpaenidae	*Scorpaenacanariensis* (Sauvage, 1878)	Mistaken	D' Oliveira (2010)	The blurred photo in D' Oliveira (2010) does not show this species
Epinephelidae	*Epinephelusaeneus* (Geoffroy St. Hilaire, 1817)	Need Confirmation	Cadenat (1935), Reiner (1996), Monteiro (1998), Monteiro (2008)	The photo in Monteiro (1998), Monteiro (2008) and the photos labelled "*E.aeneus*" in the INDP data collection actually show *Epinephelusgoreensis* (Valenciennes 1830). Cadenat (1935) explicitly notes the species not only from the African coast, but also from the Cape Verde Islands
Epinephelidae	*Mycteropercarubra* (Bloch, 1793)	Mistaken	Reiner (1996)	Called *Mycteropercarubra* (Bloch, 1793) by Monteiro (1998), Maggio et al. (2005), Medina et al. (2007) and others. A photo taken by RF at the Cape Verde Islands can be found in www.fishbase.org
Pomacentridae	*Chromislimbata* (Valenciennes, 1833)	Mistaken	Zander (2011)	Probably confusion with *Azurinamultilineata* (Guichenot 1843)
Tetraodontidae	*Lagocephaluslaevigatus* (Linnaeus, 1766)	Not valid	Lloris et al. (1991), Reiner (1996)	We can find no evidence for the presence of this species at the Cape Verde Islands

## Footnotes

*1 This is a list of the species that we consider to have a confirmed presence in Cabo Verde. There may be other species that we are not aware of or that other authors consider to be present, as well as species whose presence is not considered by other authors.
